# Clinical inertia in the treatment of heart failure: a major issue to tackle

**DOI:** 10.1007/s10741-020-09979-z

**Published:** 2020-05-30

**Authors:** Caroline Verhestraeten, Ward A. Heggermont, Michael Maris

**Affiliations:** 1 Novartis Pharma nv-sa, Medialaan 40, 1800 Vilvoorde, Belgium; 2 Cardiovascular Center OLV Aalst, Moorselbaan 164, 9300 Aalst, Belgium; 3 Cardiovascular Research Center Maastricht, Universiteitssingel 50, 6202 Maastricht, The Netherlands

**Keywords:** Clinical inertia, Guideline-directed treatment, Heart failure, Target dose, Under-treatment

## Abstract

Despite an enormous improvement in heart failure management during the last decades, the hospitalization and mortality rate of heart failure patients still remain very high. Clinical inertia, defined as the lack of treatment intensification in a patient not at evidence-based goals for care, is an important underlying cause. Clinical inertia is extensively described in hypertension and type 2 diabetes mellitus, but increasingly recognized in heart failure as well. Given the well-established guidelines for the management of heart failure, these are still not being reflected in clinical practice. While the absolute majority of patients were treated by guideline-directed heart failure drugs, only a small percentage of these patients reached the correct guideline-recommended target dose of angiotensin-converting enzyme inhibitors, angiotensin II receptor blockers, beta-blockers, mineralocorticoid receptor antagonists, and angiotensin receptor-neprilysin inhibitors. This considerable under-treatment leads to a large number of avoidable hospitalizations and deaths. This review discusses clinical inertia in heart failure and explains its major contributing factors (i.e., physician, patient, and system) and touches upon some recommendations to prevent clinical inertia and ameliorate heart failure treatment.

## Introduction

Heart failure (HF) is a common, severe, and life-threatening disease that reached epidemic proportions with an estimated 6.5 million adults currently suffering from HF in the United States (US). This number is expected to further increase to 8.5 million by 2030 [1, 2]. In the US, roughly 670,000 people are diagnosed with HF yearly and around 2% of the population is affected by HF of which 20% are hospitalized each year. Therefore, it is the leading cause of hospitalization in people older than 65 years. However, HF can no longer be considered as a problem of the elderly: the proportion of patients below 65 years who needs to be hospitalized for HF has significantly increased from 23 to 29% between 2000 and 2010 [3, 4], and there is no indication that this trend will stop. Therefore, the lifetime risk of developing HF is one in five, mainly due to an increased prevalence of the most common risk factors, such as high blood pressure, coronary heart disease, and diabetes [5, 6]. In addition, survival rates remain very low as 50% of HF patients die within 5 years after the first hospitalization, even despite improvements in HF management [2, 7–9].

HF is not only a life-threatening condition, but it is also a costly chronic disease. The high rate of hospitalizations is responsible for a massive economic burden on our healthcare systems [10]. The estimated total cost for HF in the US was $30.7 billion, a number that is expected to double by 2030. This total includes the cost of health care services, medications to treat HF, and missed days of work [2, 11].

Although there was a considerable improvement in HF management over the last 20 years, with the development of new drugs, devices, interventions, and development of extensive guidelines based on vast evidence, all accounting for decreased mortality rates, HF care is still suboptimal and mortality rates still remain unacceptably high [10]. It is clear that there is a high unmet need for new therapies. Nevertheless, the appropriate use of current HF treatments, together with a better awareness and diagnosis of the disease, can significantly contribute to improvement of patient outcomes, by lowering adverse events and mortality rate and decreasing medical care costs [9, 12].

The aim of this integrative review is to describe and better understand the different causes of clinical inertia in HF and to counteract it by raising awareness among the treating physicians and by suggesting some specific recommendations to prevent it.

## What is clinical inertia?

Clinical inertia is defined as “the lack of treatment intensification in a patient not at evidence based goals for care” [13]. However, clinical inertia is broader than failure to initiate or intensify therapy when indicated. It significantly increases the risk of adverse outcomes and raises health care costs in several chronic diseases [14]. O’Connor et al. attributed clinical inertia to three principal factors in their conceptual model: system-related factors, patient-related factors, and physician-related factors, which contribute for 20%, 30%, and 50%, respectively [13].

Clinical inertia mostly occurs in chronic diseases where patients have a limited symptom burden, leading to a higher rate of delayed and underdiagnosis. Despite the absence of severe symptoms, the disease is further progressing, making the patient at high risk to develop complications [15]. Consequently, one of the most important factors that contribute to clinical inertia are physician-related factors. Several surveys showed that patients did not receive optimal therapies, despite physicians reporting to be adherent to the guidelines [9]. One possible reason for this discrepancy is that physicians overrate the quality of care they already deliver and substantially underestimate the number of patients in need of therapy. Other potential reasons for clinical inertia are “soft excuses” due to patient non-adherence, time pressure during an office visit, and patient’s reluctance to adjust therapy [12–15]. Finally, lack of education, training, and organization are also well-identified contributors to clinical inertia [13–15].

Despite well-established guidelines for the majority of chronic diseases, these are still not reflected in clinical practice. Aujoulat et al. showed that the main reasons why physicians do not follow these guidelines are either lack of awareness of evidence-based goals of care, lack of familiarity with the guidelines, or disagreement with the guidelines [14]. Additionally, applicability of these guidelines to patients with several co-morbidities is not always evident [14]. Therefore, guidelines should be dynamic as the evidence-based practice is constantly evolving, and own clinical judgment and experience should always remain the cornerstone of treatment [16]. However, clinical judgment and experience should not preclude the correct application of guidelines, rather should these factors be of help to interpret the guidelines in a meaningful way applicable to each individual patient.

The most important patient-related factor is medication non-adherence. Adhering to medication is essential in chronic diseases, but it implies a therapeutic alliance between doctor and patient, with joint decision making and support for self-care [8]. While non-adherence is common, it is not always clinically inappropriate, e.g., medication discontinuation or reduction in dose as a result of medication side effects or intolerance could be misinterpreted as clinical inertia. This can be particularly the case in the absence of clinical information such as in the conduct of research with administrative claims [16]. Other patient-related factors include overall mistrust in and refusal of recommended treatment due to denial of (the seriousness of) a disease, delay in seeking medical care, attitude regarding medications, poor health care literacy, resistance to adopting lifestyle changes, and being unconvinced of the efficacy of the medications [12, 13].

Finally, system-related factors, such as differences between general practice and hospital-based care, lack of multidisciplinary and team-based care, lack of data to monitor quality of care and routinely identify patients in need of more intensive care, and poor communication between medical staff strongly contribute to clinical inertia [13, 14].

## Clinical inertia in HF: the elephant in the room?

### Physician-related factors

#### Adherence to the guidelines

The American College of Cardiology (ACC), the American Heart Association (AHA), and the European Society of Cardiology (ESC) all issue clear HF guidelines to support health care providers in delivering the best possible, evidence-based care to HF patients [17, 18]. These guidelines are the subject of continuous critical appraisal and are regularly updated based on new evidence. It is well demonstrated that adherence to these guidelines reduces morbidity and mortality and improves the quality of life of patients. In the ADDress your Heart study, 98% of the cardiologists admitted to be familiar with the ESC guidelines, but only 25% made treatment recommendations that exactly matched those of an expert panel, based on the ESC guidelines [9]. The main barriers to implement the guidelines in clinical practice were poor patient compliance and guidelines complexity [9]. The Heart Failure Adherence and Retention Trial (HART) showed that the combined adherence of both physicians and patients is poor as only in 41% of the cases, both physician and patient were adherent to both prescribing and taking evidence-based therapies, respectively. Physicians were deemed to be non-adherent if they failed to prescribe, in the absence of contraindications, any of the guideline-recommended medication or if they prescribed a medication in the presence of a known contraindication. Patient adherence to prescribed medication was measured by MEMS electronic pill caps, whereas patient adherence to prescribed medication was defined as taking assigned medication ≥ 80% of the time. The highest physician non-adherence rate was seen in the most vulnerable patients, i.e., those patients with a high number of comorbidities, older age, more advanced HF, and minority status [12]. Calvin et al. concluded that a better adherence towards prescribing and taking guideline-recommended medication should be a shared responsibility of physician and patient. He proposed a better HF education and awareness of the importance of an effective treatment of both physicians and patients as a potential solution to improve clinical inertia. This is in line with Komajda et al. stating that continued medical education and improved organization of services are required to improve treatment of HF in daily practice [19].

#### Under-treatment is a common problem in HF

Large international surveys, conducted in the early 2000s, showed rather good physician’s adherence to angiotensin-converting enzyme inhibitors (ACEi) (> 60%) and diuretics (> 80%), whereas the adherence to beta blockers (BB) and mineralocorticoid receptor antagonists (MRA) was much lower (30 to 60%). Although some surveys performed in European countries over the next 15 years showed an improvement in guideline adherence, only a limited percentage of patients reached the correct dosage of these guideline-recommended treatments (an overview of all the studies, registries, and surveys is found in Table 1 and will be discussed in the next paragraph) [19, 29–31].

**Table 1:** Overview of studies, registries, and surveys studying drug adherence

Study	HFrEF patients	% ACEi/ARB		% BB		% MRA		% ARNI	
		Prescribed	Target dose	Prescribed	Target dose	Prescribed	Target dose	Prescribed	Target dose
QUALIFY [20]	7092	87.2%	34.8%	86.7%	9.7%	69.3%	–	–	–
BIOSTAT-CHF [21]	2100	–	22%	–	12%	–	–	–	–
ESC HF Long-term Registry [22]	2834	92.6%	39.5%	93.3%	13.2%	75.5%	23.5%	–	–
Poelzl et al. [23]	1014	90.5%	38%	87.8%	24%	42.7%	–	–	–
TSOC-HFrEF [24]	1473	62%	5%	60%	36%	49%	21.6%	–	–
Gicc-HF [25]	275	76.3%	19%	69%	10%	–	–	–	–
CHAMP-HF [26]	3518	60.5%	17%	67%	28%	33.4%	77%	13%	14%
CHECK-HF [27]	5701	84%	43.6%	86%	18.9%	56%	52.0%	–	–
Diamant et al. [28]	370	67.3% (86.4%^a^)	22.1% (28.6%^a^)	88.4% (93.4%^a^)	30% (31.7%^a^)	38.4% (48.1%^a^)	3.2% (4.1%^a^)	–	–

^a^Eligble patients without contraindications

QUALIFY, a prospective survey analyzing physicians’ adherence to ESC guideline-recommended treatment, demonstrated that 87.2%, 86.7%, and 69.3% of patients suffering from HF with reduced ejection fraction (HFrEF) were prescribed ACEi/angiotensin II receptor blockers (ARB), BB, and MRA, respectively. The global class adherence score in the overall population was good for 67% of the patients as they received all recommended medications for their individual profile, moderate for 25% of the patients, which were receiving more than half, but not all recommended medications for their individual profile, and poor for 8% of the patients as they received ≤ 50% guideline-recommended drugs despite the absence of specific contra-indications or intolerance. Importantly, while 63.3%, 39.5%, and 51.8% of the patients received more than 50% of the target dose, only 27.9%, 6.9%, and 9.7% received the recommended target dose for ACEi, ARB, and BB, respectively [20]. Results from the ESC Heart Failure Long Term Registry, based on 2834 ambulatory HFrEF patients, showed good adherence as 92.6%, 93.3%, and 74.5% of the patients received ACEi/ARB, BB, and MRA, respectively. The true under-treatment rates, defined as the percentage of patients who, without justification, did not receive the drug, were only 3.4%, 1.8%, and 19.0%, respectively [22]. These results were further confirmed by data on 1014 ambulatory HFrEF patients from the Austrian Heart Failure Registry showing that 90.5% of the patients were treated with ACEi/ARB, 87.8% with BB, and 42.7% with MRA, but less than 70% and 50% were treated with ≥ 50% of target dose of ACEi/ARB and BB, respectively [23]. A recent publication of the CHAMP-HF registry that included 3518 HFrEF patients receiving at least 1 oral HF medication showed that among eligible patients for treatment with ACEi/ARB, BB, and MRA, only 60.5%, 67.0%, and 33.4% received the respective therapy. For angiotensin receptor neprilysin inhibitor (ARNI), the newest class for HF treatment, only 13% of the eligible patients were prescribed this lifesaving drug, despite having a class I recommendation [18]. Among patients eligible for all classes of medication, only 22.1% were simultaneously prescribed some dose of ACEi/ARB/ARNI, BB, and MRA therapy, and only 37 patients or 1.1% of the complete registry of 3518 patients were simultaneously prescribed target doses of all 3 classes of therapy. Importantly, only 1.1%, 0.2%, 1.1%, and 1.1% of all patients had contra-indications for ACEi/ARB, BB, MRA, and ARNI, respectively, whereas 39.1, 32.9%, 65.9%, and 86.1% had no contra-indication, but were not treated with ACEi/ARB, BB, MRA, and ARNI, respectively. When medications were prescribed, few patients were receiving target doses of ACEi/ARB (17%), BB (28%), and ARNI (14%), whereas the majority of patients were receiving target doses of MRA therapy (77%). As vital signs, laboratory values, ejection fractions, and the prevalence of co-morbidities were similar across all medication groups, these clinical parameters cannot account for the huge discrepancy of HF care between the studied patients [26]. When comparing the data from the contemporary CHAMP-HF registry with data from the 10-year-old IMPROVE HF registry, one can notice similar prescription rates for MRA (< 40%) and even a decrease for ACEi/ARB and BB between now and a decade ago [26, 29]. In line, the CHECK-HF registry (based on 5701 HFrEF patients) showed that in Dutch HF outpatient clinics, a high rate of patients did not receive the optimal treatment: 81% of the HFrEF patients were treated with loop diuretics, 84% with ACEi/ARB, 86% with BB, and 56% with MRA, whereas the inability to tolerate the medications was recorded in 9.4%, 3.3%, and 5.4% of the patients taking ACEi/ARB, BB, and MRA, respectively. However, even when a drug was prescribed, 24% and 45% of the patients prescribed an ACEi/ARB and BB, respectively, received less than 50% of the drug target dose. Importantly, significant differences between different dedicated HF clinics could be determined as well. Prescription rates in HFrEF patients differed significantly among the different centers: all patients received loop diuretics in one center but only 63% in another. The largest differences were seen for MRA, in which the prescription rates ranged between 34% and almost 90%. Also, the range for triple therapy (RAASi, BB, MRA) was large, from 16 to 76% [27]. In a recent study of Diamant et al., 370 hospitalized HFrEF patients were assessed: 66%, 88%, and 38% were prescribed an ACEi/ARB, BB, and MRA, respectively. Importantly, when taking into account contra-indications, 86%, 93%, and 48% were prescribed an ACEi/ARB/ARNI, BB, and MRA, respectively, in eligible patients without contra-indications. The percentage of patients prescribed ≥ 50% of target dose was 60%, 59%, and 27%, whereas the percentage of eligible patients at target dose was 29%, 32%, and 4%, for ACEi/ARB/ARNI, BB, and MRA, respectively. Among the 248 eligible patients without contraindication to any component of triple therapy, 111 (44%) received all three medication classes concurrently. Forty-two of 248 eligible patients (16.9%) were prescribed ≥ 50% of target dose, and only three patients received target dosing of all three medication classes. Frequent contra-indications to therapy included renal dysfunction, hyperkalemia, hypotension, and bradycardia [28].

#### Causes for non-prescription of guideline-recommended treatments

In the QUALIFY registry, only less than two-thirds of the patients were treated with ACEi and the main reason for non-prescription was poor tolerance (cough, hypotension, and worsening renal function in descending order of frequency). Only 21.5% of the patients were on ARB, where the main reason for non-prescription was lack of indication for such therapy, according to the investigators. BB were used in 86.7% of the patients; intolerance (36.3%) and lack of indication (35.3%) were the most common reasons for non-prescription. The main reasons for intolerance and contra-indications were worsening of asthma/chronic obstructive pulmonary disease (COPD), hypotension, and fatigue. A large proportion of patients (69.3%) were treated with MRA. Contra-indications were reported in 18.9% and intolerance in 14.9%, both mostly due to renal dysfunction or hyperkalemia [20]. This is in line with data from the ESC HF long-term registry demonstrating that the main reasons why recommended treatments were not used in patients with HFrEF were related to contra-indications and intolerance of the drugs. Most common contra-indications were severe renal dysfunction for ACEi/ARB and MRA and asthma/COPD and bradyarrhytmia for BB, whereas most common factors related to intolerance were worsening renal dysfunction for ACEi/ARB and MRA, symptomatic hypotension for ACEi/ARB and BB, and hyperkalemia for MRA [22]. In the Austrian HF registry, patients were followed for 1 year: whereas the use of ACEi was not different versus baseline (72.8% at baseline vs. 69.2% at 1-year follow-up), ARB prescription markedly increased (19.6% at baseline vs. 27.5% at 1-year follow-up). The proportion of patients on BB also increased over time (87.8% at baseline vs. 91.6% at 1-year follow-up), whereas the prescription of MRA declined (42.7% at baseline vs. 38.9% at 1-year follow-up). Interim hospitalization for worsening HF, ischemic cardiomyopathies, renal impairment, and older age was associated with the reluctance of treating physicians to improve guideline adherence over time, whereas higher N-terminal pro b-type natriuretic peptide (NT-proBNP) and higher systolic blood pressure at baseline favored an improvement in therapy [23]. In the TSOC-HFrEF registry, old age was universally associated with non-prescription of each class of guideline-recommended therapies. Higher serum creatinine level was associated with nonprescription of ACEi/ARB and MRA, whereas asthma or COPD was associated with non-prescription of BB. However, COPD is not an absolute contraindication for BB use unless worsening symptoms develop after BB treatment. The registry could not elucidate the fact whether the under-usage of BB was due to deterioration of pulmonary condition or the fear of side effects. In general, the authors concluded that physician-related factors for non-prescription of guideline-recommended therapies could be related to the fear of adverse events that can occur during initiation or dose escalation of guideline-recommended medications. However, as the registry did not record the reason of non-prescription, and as the percentage of adverse events was unknown, it is difficult to make a bold statement, e.g., it was observed that the prescribing rate of ACEi decreased over time whereas that of ARB increased from hospital discharge to 1-year follow-up. This trend could be explained by patients not tolerating side effects of cough or angioedema from ACEi and had therapy switched from ACEi to ARB [24]. An overview of the described studies, registries, and surveys mentioning the main causes for non-prescription of guideline-recommended treatments is found in Table 2.

**Table 2:** Overview of studies, registries, and surveys studying the main causes for non-prescription of guideline-recommended treatments

	QUALIFY [20]	ESC HF Long-term Registry [22]	TSOC-HFrEF [24]
ACEi/ARB	Worsening renal function	Worsening renal function	Worsening renal function
	Hypotension	Hypotension	
	Cough		
			Older age
BB	Worsening of asthma and COPD		Worsening of asthma and COPD
	Hypotension	Hypotension	
	Bradycardia		
	Fatigue		
		Bronchospasm	
			Older age
MRA	Hyperkalemia	Hyperkalemia	
	Renal dysfunction	Renal dysfunction	Renal dysfunction
			Older age

#### Causes for not reaching target doses of guideline-recommended treatments

The BIOSTAT-CHF, a European trial specifically designed to study the up-titration of ACEi/ARB and/or BB in 2100 HFrEF patients demonstrated that during an up-titration period of 3 months, only 22% and 12% of patients achieved the recommended treatment dose for ACEi/ARB and BB, respectively. Independent predictors for achieving lower percentages of recommended ACEi/ARB dose were female sex, country of inclusion, lower BMI, and estimated glomerular filtration rate (eGFR). Predictors for lower BB doses were higher age, country of inclusion, lower heart rate diastolic blood pressure, and more signs of congestion (all *P* < 0.05). Additionally, patients not reaching BB dose were somewhat older (*P* = 0.08), were longer diagnosed with heart failure (*P* = 0.07), had more atrial fibrillation (AF) (*P* = 0.06), and lower diastolic blood pressure (DBP) (*P* = 0.08). Marked differences in dose-uptitration were found across Europe as lower ACEi/ARB and BB doses were achieved in South and Central European countries, while Scandinavian countries achieved higher ACEi/ARB and BB doses [21]. In the ESC Heart Failure Long Term Registry, target doses were only reached in 39.5%, 13.2%, and 23.5% for ACEi/ARB, BB, and MRA, respectively. There was often a clinical reason indicating that the dose prescribed was optimal for the patient, but in at least 1 of 4 patients (1 of 2 in the case of MRA), no justification was recorded. Most important reasons why target dose was not reached were patients still being in the titration phase (> 25% for ACEi/ARB/BB/MRA), symptomatic hypotension (> 30% for ACEi/ARB and 20% for BB), hyperkalemia (10.4% for MRA), and bradyarrhythmia (9% for BB) [22]. In line, the TSOC-HFrEF registry, a prospective survey of HFrEF patients hospitalized due to acute decompensation, showed that at discharge, 62%, 60%, and 49% of these patients were prescribed ACEi/ARB, BB, and MRA, respectively. The proportions of patients reaching ≥ 50% of the target dose were 24.4%, 20.6%, 86.2%, respectively, and only 5%, 3.6%, 21.6% reached maximal dose levels. At 1-year follow-up, dosages of ACEi/ARB and MRA were up-titrated in about one-fourth of these patients, whereas dosages of BB were up-titrated in about 40% of the patients. Nonetheless, the proportion of patients reaching ≥ 50% of the target doses was rather similar as compared to discharge, meaning that the physician did not further improve the care of the HF patient at follow-up visits leaving these patients at augmented risk [24]. Similar findings were noted in the recent Gicc-HF study collecting clinical data and medications of 275 HFrEF patients during hospitalization and 3 months post-discharge. Between admission and discharge, usage of ACEi and BB increased by 19 to 20% and MRA by 8%. At discharge, ACEi or ARB were prescribed in 80% of cases with the mean dose reaching 36 ± 31% of target dose, BB in 70% with the mean dose of 27 ± 51% of the target dose and MRA were prescribed in 23% of cases. Three months after discharge, there were few changes in medications. Initiation of ACEi or ARB, BB, and MRA was performed in 3 to 7% while cessation was performed in 5 to 6% of cases. Changes in doses were observed in about 25% of all cases. At 3 months after discharge, the usage of ACEi/ARB ≥ 50% of target dose was significantly related to usage of ACEi/ARB at discharge (OR 5.67), age (OR 0.97), and the creatininemia at discharge (OR 1.0263). The usage of BB ≥ 50% of target dose was significantly related to usage of BB at discharge (OR 4.22) and COPD (OR 0.37) [25]. The post-discharge period has been called the ‘vulnerable phase’ of HF because of the very high risk of unplanned readmission or death. Therefore, a close follow-up of the patient, implementation of new medication, and up-titration of life-saving drugs to the right dose is key in this time window to avoid subsequent hospitalizations [32, 33]. This is supported by the findings of Verbrugge et al. showing that HFrEF patients who were up-titrated with ACEi or BB during or immediately after hospital admission had significant reductions by 64% and 49%, respectively, in the composite end-point of all-cause mortality or HF [34]. In line, continuation of guideline-recommended drugs among patients hospitalized for HFrEF was associated with significantly lower mortality and hospital readmission, as compared to those who were discontinued; so, the in-hospital setting provides a key opportunity to re-address and optimize current medical therapy [35]. An overview of the described studies, registries, and surveys mentioning the main causes for not reaching target doses of guideline-recommended treatment is found in Table 3.

**Table 3:** Overview of studies, registries, and surveys studying the main causes for not reaching target doses of guideline-recommended treatments

	BIOSTAT-HF [21]	ESC HF Long-term Registry [22]	GICC-HF [25]
ACEi/ARB	Female sex		
	Lower BMI		
	eGFR		
		Hypotension	
			Older age
			Worsening renal function
BB	Higher age		
	Lower heart rate		
	Lower diastolic blood pressure		
	More signs of congestion		
		Hypotension	
		Bradyarrhytmia	
			Presence of COPD
MRA		Hyperkalemia	

#### Adherence to guideline-recommended treatments leads to improved clinical outcomes

From these data, all pointing in the same direction, it is clear that exclusive use of the percentage of patients treated by guideline-recommended drugs is a poor indicator of the quality of healthcare in HF. Measures should be taken to improve the attainment of optimal dosing in each patient. Moreover, there is overwhelming evidence that higher doses of guideline-recommended drugs are associated with improved outcomes. QUALIFY clearly demonstrated that poor adherence to guideline-directed treatment, defined as use of < 50% of target doses, was associated with significantly higher overall mortality (HR 2.21) and cardiovascular mortality (HR 2.27) as compared to good adherence, defined as use of ≥ 50% of target dosage [36]. These results were corroborated by the BIOSTAT-HF trial, showing a significantly higher mortality (HR 1.76 and 2.41) in patients that reached < 50% of the recommended ACEi/ARB or BB dose, respectively, as compared to patients treated on target levels. Patients not reaching recommended dose because of symptoms, side effects, and non-cardiac organ dysfunction had the highest mortality rate. For ACEi/ARB, the HR for not reaching recommended dose because of symptoms, side effects, and non-cardiac organ dysfunction was 1.72 and 1.46 for ‘other reasons’. Not reaching the recommended dose of BB because of symptoms, side effects, and non-cardiac organ dysfunction was associated with an increased mortality risk (HR 1.70) while the mortality risk was not increased in patients who did not reach the recommended dose for ‘other reasons’ (HR 1.18) [21]. In line, the Norwegian Heart Failure Registry demonstrated that after treatment optimization of HFrEF patients, 89.8% were indeed treated with an ACEi/ARB, but only patients that reached ≥ 50% of the maximal recommended target dose for ACEi had a significantly better survival (HR 0.65 for all-cause mortality) [37]. In the Austrian HF registry, it is important to notice that optimization of guideline adherence was paralleled by a decrease in disease severity and resulted in a significant reduction in all-cause mortality risk. More detailed improvements in the guideline adherence indicator (GAI) and GAI50+ were associated with significant improvements in NYHA classification and NT-proBNP. Improvements in GAI50 were also independently predictive of lower mortality risk (HR 0.55 [95% CI 0.34–0.87; *p* = 0.01]) after adjustment for a large variety of baseline parameters and hospitalization for HF during follow-up [23]. An overview of the described studies, registries, and surveys mentioning the influence of good versus poor adherence to guideline-recommended treatments on clinical outcomes is found in Table 4.

**Table 4:** Overview of studies, registries, and surveys studying the influence of adherence on clinical outcomes

QUALIFY [36]^a^	BIOSTAT-HF [21]^b^	Norwegian Heart Failure Registry [37]^a^	Austrian HF registry [23]^a^
All-cause mortality (HR 0.45)	All-cause mortality (HR 0.57) for ACEi/ARB	All-cause mortality (HR 0.65)	All-cause mortality (HR 0.55)
CV mortality (HR 0.44)	All-cause mortality (HR 0.41) for BB		
HF mortality (HR 0.44)			

^a^Poor adherence: defined as use of < 50% of target doses; good adherence: defined as use of ≥ 50% of target dosage

^b^Poor adherence: defined as use of < 50% of target doses; good adherence: defined as use of 100% of target dosage

#### Limitations

While non-adherence is common, not all non-adherence or non-persistence is clinically inappropriate and therefore cannot be classified as clinical inertia. Particularly, medication discontinuation or reduction in dose as a result of medication side effects or intolerance should not be considered as clinical inertia. Additionally, clinical inaction may also reflect appropriate care in certain circumstances, such as prior visits with satisfactory readings, side effects from previously prescribed drugs that preclude new options, and patients’ informed preference against intensified treatment [38]. When using administrative claims, registries, and databases, there is often a lack of data on medication dose leading to underestimation of true treatment intensification, which would include dose escalation. Also, adverse events or contra-indications, important reasons why up-titration or medication addition are lacking, can often not be found in these registries and this might create a bias. Finally, medication addition may have benefits over dose escalation including higher efficacy and fewer side effects [16, 38].

## Patient-related factors

Patients also play an important role in clinical inertia. A meta-analysis clearly showed that the effect of medication adherence interventions in HF patients reduces the mortality risk by 10.6% [3], whereas not only medication, but also lifestyle adherence is essential. Treatment adherence can be improved by active participation of patients in the context of shared decision making and by developing realistic expectations of the disease course [8]. Besides medication non-adherence, there is also a lack of awareness of HF among the public in Europe. The SHAPE survey, designed to map public recognition of HF, demonstrated that awareness of HF is low in Europe, and therefore, patients are unlikely to demand appropriate measures by healthcare authorities and providers [6]. Giezeman et al. reported that there is still room for improvement in patient education and self-care behavior, as participants who were treated and followed up in a HF clinic had a significantly better self-care behavior [39]. In line, a study investigating the impact and experience of using an interactive patient website designed to give patients individual feedback about their condition and to suggest tailored questions for patients to ask their physician showed that patients who used the website had a positive shift in their attitudes regarding interactions with their physicians. Use of the website also prompted patients to become more actively involved in their disease care [40].

## System-related factors

Next to physician- and patient-related factors, system factors play an important role in clinical inertia. With the increased burden of several chronic diseases, health care systems will have to introduce new approaches, such as adapted organizational structures with the involvement of general practitioners and specialized nurses. Key points contributing to tackle clinical inertia are an integrated multidisciplinary care, specialized care paths and centers, and a proper well-organized follow-up of the patients. GPs play a key role in HF management, but despite multiple guidelines, the management of patients with HF in primary care is suboptimal. A Belgian study showed that GPs expressed the need for a multidisciplinary chronic care approach for HF. Currently, waiting lists and the poor local availability of specialized care, such as cardiology services, open-access echocardiography, HF clinics, and HF nursing teams, had a negative effect on GPs’ decisions to refer patients with suspected HF, leading to delayed diagnosis and suboptimal care [41]. The EUROACTION trial, a randomized, controlled trial in eight European countries, examined the effect of a nurse-coordinated multidisciplinary, cardiovascular disease prevention program over 1 year as compared with usual care. The results show that there was significant attainment of the blood pressure target, significant reduction in cholesterol for higher-risk patients, a higher prescription rate for statins in hospitalized patients, and a better diet consumption in the nurse-coordinated group [42]. In addition, there is also a place for pharmacists. Schulz et al. demonstrated in a small trial of 110 HF patients that a pharmacy-based intervention, including medication review, regular dose dispensing, and counseling, improved mean adherence to three HF medication classes, increased the proportion of adherent patients, and led to clinically important improvements in quality of life [43].

Results from IMPROVE HF clearly showed a significant improvement in adherence to guideline-recommended care among practices 24 months after implementation of a performance improvement intervention. This intervention consisted of the use of decision support tools, patient data collection, and performance feedback, concentrating on the specific therapies proven to improve outcomes [29]. The GUIDE-IT trial was designed to determine whether a NT-proBNP-guided treatment strategy improves clinical outcomes compared to usual care in high-risk HFrEF patients. This trial showed that only 31% and 15% of the patients reached target doses of ACEi/ARB and BB, respectively, despite care guided by natriuretic peptide levels and a median 12 clinic visits and 6 adjustments to HF treatments over a median 15-month follow-up period. Therefore, it could be concluded that a strategy of NT-proBNP-guided therapy was not more effective than a usual care strategy in improving outcomes, such as cardiovascular mortality and hospitalization for HF in HFrEF patients [44]. It has been shown that educational interventions directed towards physicians, addressing specific care needs, led to improved care as well. It has been suggested that local opinion leaders, highly respected by their colleagues, can help educate and promote primary care practitioners. In this view, one study of a primary care faculty showed a tendency toward an inverse relationship between knowledge of guidelines and hypertension control [42].

Using an integrated model for HF patients, where monitoring of chronic patients can be redistributed between primary care and cardiology, could lead to improved satisfaction levels and intensified treatment without any increase in use of resources [45]. An important example of integrated care is HF clinics. An analysis of 18 randomized studies comparing HF clinics with conventional care showed either a reduction in hospital readmissions or shortening of hospitalizations in the intervention group in the majority of studies [46, 47]. A study including 8792 patients examined the long-term adherence to and dosages of evidence-based pharmacotherapy during and after participation in specialized HF clinics. Adherence to evidence-based HF treatment is high, with 95% and 88% of patients attending specialized HF clinics receiving renin-angiotensin system (RAS) inhibitors and BB, respectively. Adherence remained high in patients referred back to their GP for long-term follow-up: 89%, 89%, and 72% of the patients were still taking RAS inhibitors, BB, and MRA, respectively, 1 year after leaving the HF clinic. It is reassuring to learn that the effect on patient medication behavior gained during HF clinics follow-up is maintained after the patients return to their GP for long-term follow-up. It suggests that patients received adequate education during their visits to the HF clinics, enabling patients to manage their own disease. It also suggests that GPs receive adequate information from the HF clinics about the treatment plan and that the GPs cooperate with this plan [48].

Some studies have shown that interventions (e.g., periodic monitoring of symptoms/signs and reviews of pharmacological therapy) aimed at improving the management of patients with HF after hospital discharge are correlated with a significant decrease in hospital readmission rates. However, the heavy economic costs related to the systematic organization of patient follow-ups after hospital discharge have pushed the development of remote monitoring systems for the continuous control of clinical variables. Results of several studies have demonstrated that telemonitoring has beneficial effects on clinical outcomes of HF including a reduction in mortality, HF hospitalization, all-cause hospitalization, and an improvement in quality of life. Therefore, it can be concluded that key elements of telemonitoring including physiological monitoring of blood pressure, heart rate, weight, and electrocardiogram (ECG) must form an integral part of the routine care of patients with HF [49, 50]. Despite clear advantages, the clear-cut reimbursement restriction of telehealth services is a big hurdle to their dissemination. Additionally, the replacement of traditional face-to-face evaluations with digital ones (absence of doctor-patient relationship) and the fragmentation of care that would probably be delivered by heterogeneous and non-interconnected professionals may result in patients receiving different and possibly conflicting recommendations for identical clinical pictures [51].

## Conclusion and outlook

A vast amount of scientifically sound data, mainly collected by double-blind randomized controlled clinical trials (RCTs), supports the use of several oral medications and devices for HF patients, whereas more advanced therapies are available for patients with advanced HF. All these therapeutic options have been summarized by expert groups of the main cardiovascular associations (AHA, ACC, ESC) and made available in easy-accessible and ready-to-use schemes [17, 18]. We have numerous tools available to treat HF patients (at least HFrEF patients) in the most optimal way, but nevertheless, HF is still a common, serious, progressive, costly disease with high mortality rates [52]. This statement can be supported by numerous prospective and retrospective data (“real life data”), where it could be observed that the mortality rate does not improve substantially as expected based on RCT data. Furthermore, studies specifically designed to monitor if patients are on the indicated treatments, and/or on the target dose of those, show dramatic results: a rather low percentage of patients are treated by all indicated therapies, and the majority of these patients do not reach target doses of the guideline-recommended treatments. The central question remains: why do we observe a massive under-treatment of these vulnerable patients? In this review, we summarized data to show that clinical inertia is one of the main factors leading to this under-treatment. Taken together, clinical inertia is a multifactorial phenomenon with several causes and it occurs many times within complex clinical situations. Consequently, development of interventions to reduce clinical inertia in HF is not always obvious and a multifactorial approach, focusing on the physician, the patient, and the office structure will be the best strategy to optimize the effectiveness of HF management [13].

A cornerstone to tackle the problem of clinical inertia is to make sure that all physicians acknowledge the relevance and the problem of clinical inertia. Increased awareness among HF specialists should lead to strategies to alleviate, or abandon, clinical inertia. The authors propose the following ready-to-use advice in order to tackle the major problem of clinical inertia in HF:

- at every ambulatory visit of your HF patient, ask yourself: is my patient treated with *all* mandatory medications with respect to her/his classification of HF?
  - if yes: is the *dose* of these medications titrated to the optimum (i.e., the highest tolerated dose)
  - if not: why not, and consider the options to *increase the dose* or *add a class* of guideline-directed treatment
- in the framework of a hospitalization due to decompensated HF, ask yourself the following questions:
  - what is the reason for this decompensation, and could a *problem with medication* contribute to the decompensation?
  - is my patient treated with *all* mandatory medications with respect to his classification of HF?
    - if yes: is the *dose* of these medications titrated to the optimum (i.e., the highest tolerated dose)
    - if not: why not, and consider the options to *increase the dose* or *add a class* of indicated therapy
- prone your patient to the reasoning that the *up-titration of HF medication is an obligation with beneficial effect on morbidity and mortality*, and does not mean that the patient is not doing well (start with this as early as possible in the disease process, preferably at diagnosis)
- at every HF staff meeting, when a *holistic approach* of the HF patient is discussed, the *medication scheme* should be critically assessed
- HF physicians should *listen to the HF nurse*, closest ally of both patient and physician: there might be legitimate reasons for not taking specific HF medications (e.g., important side effects, intolerance, …)
- starting, interrupting, permanently stopping, or changing the dose of medications should be carefully *noted and dated in the medical file*, including specific side effects
- provide your patient with a comprehensive ‘manual for living with HF’, explaining the disease in layman’s terms yet stressing the role of medical treatment optimization
- identify potential side effects of medication and explain them in a nuanced way (e.g., symptomatic hypotension is different from a perceived low blood pressure upon automatic pressure monitoring if the patient feels well)

Key factors to success are multidisciplinary teams assessing the status of HF patients, including their medication scheme. Also, patients need to be informed and convinced from the diagnosis of their disease onwards that adding and up-titration of medication is not because they are clinically deteriorating, but to the contrary, i.e., that add-on therapies will prevent mortality and readmissions and will improve quality of life in the longer term. HF nurse-led clinics, with a central role for the nurse in the assessment of patient status, and thereby creating an environment for a critical appraisal by the HF nurse of the current therapy initiated by the HF physician, will promote a dynamic adaptation of the medication scheme and will trigger awareness for clinical inertia. Also the patient should be kept informed, educated, and motivated to be adherent to the therapy. From the studies discussed in this review, it becomes apparent that the most vulnerable patients, i.e., these with multiple comorbidities, are at increased risk for suboptimal treatment and this should alarm HF physicians to pay special attention to this subgroup of patients.

In conclusion, the importance of clinical inertia in HF is increasing and substantiated by a tremendous amount of data. Continuous awareness, and active strategies to prevent inertia, in a multidisciplinary approach, thereby centralizing the patient with HF, will help to mitigate this phenomenon and will improve the prognosis of HF patients. Nevertheless, further research is warranted to develop sustainable strategies to eradicate clinical inertia.
